# Social representations of nurses of the Emergency Care Unit towards people with mental disorder^*^

**DOI:** 10.1590/1980-220X-REEUSP-2022-0298en

**Published:** 2023-03-13

**Authors:** Anna Carla Bento Sabeh, Hellen Pollyanna Mantelo Cecilio, Claudinei José Gomes Campos, Helca Franciolli Teixeira Reis, Anneliese Domingues Wysocki, Edirlei Machado dos Santos

**Affiliations:** 1 Universidade Federal de Mato Grosso do Sul, Três Lagoas, MS, Brazil.; 2 Universidade Estadual de Campinas, Faculdade de Enfermagem, Campinas, SP, Brazil.; 3 Universidade Federal da Bahia, Vitória da Conquista, BA, Brazil.; 4 Universidade Federal de São Paulo, Escola Paulista de Enfermagem, São Paulo, SP, Brazil.

**Keywords:** Mental Healt, Psychiatric Nursin, Emergency Medical Service, Mental Health Assistanc, Nursing Care, Salud Mental, Enfermería Psiquiátrica, Servicios Médicos de Urgencia, Atención a la Salud Mental, Atención de Enfermería, Saúde Mental, Enfermagem Psiquiátrica, Serviços Médicos de Emergência, Assistência à Saúde Mental, Cuidados de Enfermagem

## Abstract

**Objective:**

To understand the Social Representations of nurses from an Emergency Care Unit about the care provided to people with mental disorders.

**Method:**

Qualitative exploratory study, supported by the theoretical and methodological framework of the Theory of Social Representations. Interviews were conducted from July to August 2021. The data were processed using the IRaMuTeQ software.

**Results:**

22 nurses were interviewed. From the processed data, three discursive categories were constructed: the tortuousness of the Psycho-Social Care Network (RAPs): in search of a path; the abyss between the health professional and the person with mental disorder; and unveiling the care of nurses in the Mental Health Emergency Care Unit.

**Conclusion:**

We identified a lack of knowledge about the Psychosocial Care Network, anchoring elements associated with previous negative experiences that influence the professional-patient relationship, and nursing care permeated by cognitive barriers. Such findings are unprecedented in the locality studied and relevant to promote the qualification of the work of nurses in mental health in the Emergency Care Unit.

## RESUMO

**Objetivo:**

Compreender as Representações Sociais de enfermeiros de uma Unidade de Pronto Atendimento sobre o cuidado dispensado às pessoas com transtornos mentais.

**Método:**

Estudo exploratório qualitativo, sustentado pelo referencial teórico-metodológico da Teoria das Representações Sociais. Foram realizadas entrevistas no período de julho a agosto de 2021. Os dados foram processados com o auxílio do software IraMuTeQ.

**Resultados:**

Foram entrevistados 22 enfermeiros. A partir dos dados processados foram cosntruídas três categorias discursivas: a tortuosidade da Rede de Atenção Psiscossocial: em busca de um caminho; O abismo entre o profissional de saúde e a pessoa com transtorno mental; e desvelando o cuidado dos enfermeiros da Unidade de Pronto Atendimento em saúde mental.

**Conclusão:**

Identificou-se falta de conhecimento sobre a Rede de Atenção Psicossocial, elementos de ancoragem associados à experiências prévias negativas que influenciam a relação profissional-paciente e, um cuidado de enfermagem permeado por barreiras cognitivas. Tais achados são inéditos na localidade estudada e relevantes para promover a qualificação do trabalho do enfermeiro em saúde mental na Unidade de Pronto Atendimento.

## RESUMEN

**Objetivo:**

Conocer las Representaciones Sociales de las enfermeras de una Unidad de Cuidados de Urgencia sobre los cuidados prestados a personas con trastornos mentales.

**Método:**

Estudio exploratorio cualitativo, apoyado en el marco teórico y metodológico de la Teoría de las Representaciones Sociales. Las entrevistas se realizaron entre julio y agosto de 2021. Los datos se procesaron con el software IraMuTeQ.

**Resultados:**

Fueron entrevistadas 22 enfermeras. A partir de los datos procesados, se construyeron tres categorías discursivas: lo tortuoso de la Red de Atención Psicosocial: en busca de un camino; el abismo entre el profesional de la salud y la persona con trastorno mental; y develar el cuidado de las enfermeras en la Unidad de Atención de Emergencias en Salud Mental.

**Conclusiones:**

Identificamos un desconocimiento de la Red de Atención Psicosocial, elementos de anclaje asociados a experiencias negativas previas que influyen en la relación profesional-paciente, y cuidados de enfermería permeados por barreras cognitivas. Tales hallazgos son inéditos en la localidad estudiada y relevantes para promover la cualificación del trabajo de enfermería en salud mental en la Unidad de Cuidados de Urgencia.

## INTRODUCTION

The Brazilian Psychiatric Reform (BPR) proposes a collectively produced project of change in the model of attention and management of mental health care, replacing an asylums-based mental health model by a model of community services with strong territorial insertion^(1)^. Such movement had as target population people with severe and persistent mental disorders, in a greater situation of vulnerability and segregation, from which in the present research the mental health care is related to these patients. From this perspective, the Psychosocial Care Network (RAPs in the Portuguese acronym) was established, whose purpose is the creation, expansion and articulation of health care points for people with mental disorders^(2)^.

The RAPs is characterized by the diversification of care points and possibilities of arrangements for interventions in mental health^(3)^. In the scope of urgency and emergency care, this decentralization of care ensured that the reception of psychiatric urgency and emergency situations were no longer exclusive to health services specialized in mental health, which meant that the general urgency and emergency services also absorbed this demand.

A study^(4)^ verified the predominance of unpreparedness of the teams of these services and of the places of attendance in these situations. Professionals dispute about the responsibility of emergency departments in attending people with mental disorders^(5–7)^, even claiming that the psychiatric hospital is the best direction for this type of demand^(8)^. There is a significant lack of knowledge about the flows and components of the RAPs and the non-recognition of urgency and emergency services as an integral part of this network^(9)^.

Based in the conceptions presented, there is an emerging need to understand which cognitive elements (images, concepts, categories, theories) are present in health professionals about mental health care in general urgency and emergency services and about the RAPs. For this, we used the Theory of Social Representations (TSR), which is a modality of practical knowledge, directed to communication and to the understanding of the social context in which we live. Thus, it is presented from forms of knowledge that reveal themselves as cognitive elements, which allow modeling what is given from the outside, from the relationships that individuals and groups constitute with the objects, acts and situations in social interactions^(10)^, in this study focused in the mental health care to people with mental disorders. Two fundamental processes stand out in the formation of Social Representations (SR), namely objectification and anchoring, the first being responsible for giving a representation the status of objective reality and the second for integrating and categorizing new information that will guide the understanding and action^(11)^.

Using these concepts, this study aimed to understand the SR of nurses of an Emergency Care Unit (UPA in the Portuguese acronym) about the care given to people with mental disorders.

## METHODS

### Study Design

This is a study with a qualitative methodological approach, with an exploratory focus, having TSR as its theoretical and methodological reference. Such theoretical contribution was coined by Serge Moscovici in 1961, and is characterized as a form of socially elaborated and shared knowledge, which contributes to the construction of a common reality for a social group, having importance in social life and in the elucidation of cognitive processes and social interactions^(11)^.

The recommendations of the Consolidated Criteria for Reporting Qualitative Research (COREQ) guide were followed for the construction of this study^(12)^.

### Population

Twenty-two nurses who are part of the staff Emergency Care Unit (UPA) participated in this study.

### Setting

The research setting was a UPA in Três Lagoas, Mato Grosso do Sul, Brazil.

### Selection Criteria

Direct healthcare nurses of both genders with at least one year of experience in the UPA participated in the study. They agreed to participate individually, voluntarily and free of any coercion. Nurses who performed exclusively administrative activities were excluded.

### Sample Definition

The exhaustion criterion^(13)^ was used, in which all eligible subjects were approached.

### Data Collection

Data were collected in July and August 2021, by means of interviews. For this purpose, a semi-structured script was used, composed of two sections: the first containing quantitative variables aimed at obtaining sociodemographic/professional characteristics, and the second composed of qualitative questions with the purpose of extracting the SRs of the nurses regarding their performance in mental health and about the RAPs.

The interviews were conducted by the responsible researcher, in the UPA, during the professionals’ breaks, ensuring that the environment was reserved and provided privacy to them, respecting the health measures in place due to the Covid-19 pandemic.

The interviews lasted about an hour and the content was recorded in the data collection instrument itself and through audio recording, using a device for this purpose, with the consent of the participants. Afterwards, the speeches were transcribed in their entirety.

### Data Analysis and Treatment

The data were collected into a Microsoft Word document, then transferred to a Notepad and organized to be processed by the software IRaMuTeQ (Interface de R pour les Analyses Multidimensionnelles de Textes et de Questionnaires) version 0.7 alpha 2, developed by Pierre Ratinaud in 2009, in order to build the text corpus.

Among the different processing possible through IRaMuTeQ, we used the Descending Hierarchical Classification (DHC), which classifies Text Segments (TS) according to their respective vocabularies and divides them according to the frequency of reduced forms, generating a dendogram that illustrates the relationships between these classes^(14)^.

In this processing, a total of 794 TS were obtained, of which 642 were classified, generating a utilization of 80.86% and a subvision in 6 classes. In this study, we considered the first 20 most significant evoked words in the classes, and the same subsidized the construction of three categories that portray the representational contents of nurses about the theme of the study.

Therefore, the categorical analysis unfolded from the classes processed by IRaMuTeQ, which considers the most relevant lexicons in relation to the processed text corpus. Thus, the software presents the TSs that are representative for the selected lexicons in the process of building the classes derived from the DHC.

### Ethical Aspects

The study followed all the guidelines and legal prerogatives established by Resolutions 466/2012 and 510/2016, of the National Health Council. It was cleared by the Research Ethics Committee of the Federal University of Mato Grosso do Sul by Opinion No. 4.555.902/2021. All participants were instructed and clarified about the study, and given the Informed Consent Form (ICF), which was duly signed after acceptance to participate in the research. The confidentiality and anonymity of the participants was guaranteed through the use of the codename “Interviewee” followed by a number indicating the sequence in which the interviews were conducted.

## RESULTS

Twenty-two nurses participated in the study: 16 (72.7%) were female and 6 (27.3%) were male; ages ranged from 26 to 58 years; average time of training in nursing was 12.5 years.

The professionals were mostly graduated in public institutions (n = 14; 63.6%); 12 (54.5%) reported not having had access to the necessary knowledge about mental health during their graduation. As for complementary education, 7 (31.8%) had completed a second undergraduate course, 21 (95.4%) had at least one finalized latu sensu specialization, and 3 (13.6%) had a strictu sensu postgraduate course in progress (academic master’s degree).

As for specific knowledge in mental health, 1 (4.5%) reported specialization in this area, still in progress; 8 (36.4%) had taken some course related to mental health, but half (n = 4; 18.2%) more than 3 years ago. The great majority of professionals (n = 21; 95.4%) recognize the need to provide mental health care in the UPA, and all of them (n = 22; 100%) affirm that they face this kind of care frequently.

**Figure 1.**
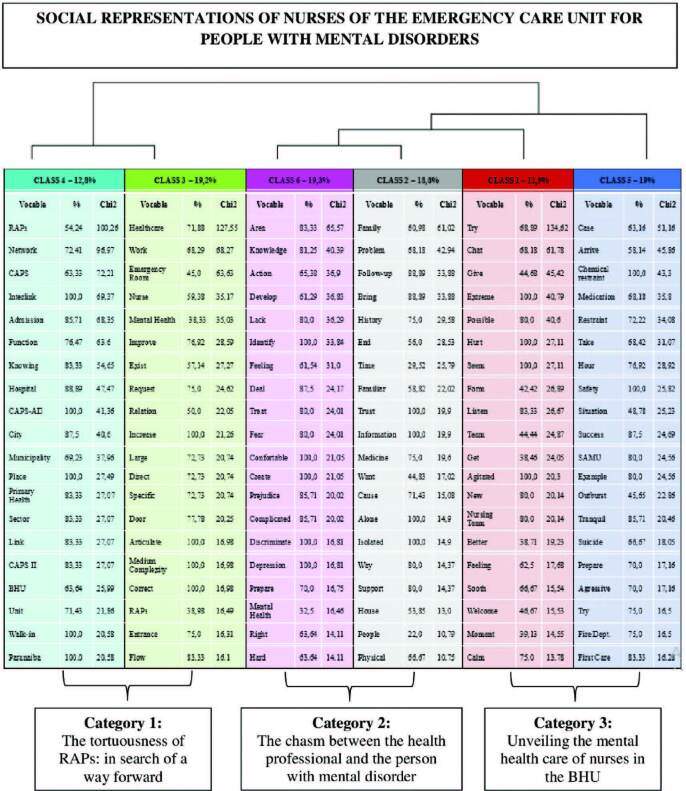
Classification of vocabulary and division of categories.

The processing was based on the lexicons identified as significant in the text corpus, which were extracted from the TS from the interviews, from which the excerpts were representative and relevant for each class extracted. Then, they were organized in the form of a dendogram, from which a categorical analysis was performed, which resulted in the identification of three categories according to the similarities identified among the classes: a) “The tortuousness of the RAPs: in search of a path”; b) “The chasm between the health professional and the person with mental disorder”; and c) “Unveiling the mental health care of nurses in the UPA” (Figure 1).

### Category 1: The Tortuousness of the RAPs: in Search of a Path

This category emerged from the lexicons coming from classes 3 and 4. It is observed that the RAPs is configured as an unknown element for the research participants. This gap objectifies the social representation about a mental health care in the distant Primary Care Unit (PCU), since what sustains the social representation are elements related to the weaknesses about the role of the PCU as a component of the PAR. The following TSs illustrate such aspects:

*I don’t even know. Nothing* (…)*. Never heard of it, I’ll even research it. I didn’t know that there was this network* (Interviewee 12).

*No, I can’t tell you anything* (…) (Interviewee 18).

*(‘) I don’t know how the city’s Psychosocial Network is working, I am kind of lost related to this* (Interviewee 16).

The incipient recognition of the PCU as part of the RAPs can have a significant impact on the work and, therefore, on the way nurses perform their daily practice, specifically about mental health care. Thus, it is observed that some participants try to bring to the familiar something unknown in their consensual universes:

*Well, the Psychosocial Care Network covers as much mental health as people who use alcohol and drugs, who have this kind of dysfunction.* (…)*. An awareness is being created in the municipality, but in a slow pace. One thing at a time* (Interviewee 13).

*Currently I see that it has improved. There is still an articulation, in a loose way, a little lost* (…) (Interviewee 19)

Furthermore, some professionals base their SR on structural weaknesses of the emergency service to justify the way they develop their mental health care to users arriving at the ED:

(…) *we have a minimal structure to attend this staff. We see that there is a high demand and these people are unassisted in the continuity of treatment* (…)*. For us, the patient is kind of left out here* (…) (Interviewee 05)

(…)*, in my point of view, I think that patients are staying too long in the Emergency Room and, in reality, it is not an ideal place* (…)*. We feel that it is impossible. We don’t have a physical apparatus to hold this patient inside the unit.* (Interviewee 20)

From the previous objectivation and anchors, one perceives a transfer of responsibility from what is produced in relation to mental health to the ills associated with how the flow of care occurs and the structure of the service.

### Category 2: The Chasm Between the Health Professional and the Person with Mental Disorder

This category originated from the lexicons derived from classes 6 and 2, in which questions were extracted about the representation/significance of mental health care in the work of nurses in the UPA, and what are the feelings perceived by professionals during these sessions. The lack of identification and affinity with the mental health area was widely verbalized:

(…) *it is not an area that I identify myself, but we have to attend. I consider it a very complex area, very difficult* (…)*. We get mentally exhausted* (…) (Interviewee 01)

(…) *it is a type of urgency in the UPA that I do not feel comfortable attending. It is one of the things that if I could choose, obviously we do not have this option, but I would prefer not to attend this type of situation* (…) (Interviewee 14)

*Mental health has never been my strong suit. Particularly, I do not have much affinity with mental health nursing* (Interviewee 11).

It is observed that the practice of mental health care is represented as something that has its own complexity and superior to other areas, causing discomfort and triggering negative feelings in professionals. Such feelings are expressly verbalized by another participant:

*Fear! I’m afraid because I’ve already been assaulted by a mental health patient, a disorder* (…)*. If there is another colleague to attend to I prefer it.* (Interviewee 08)

It is identified that the feeling of fear reported by the professional is anchored in previous unsuccessful experiences in these types of care, and that such a situation caused a permanent fear of the professional to attend patients with mental health needs. Difficulties related to lack of preparation and lack of flows and routines for this type of care were also verbalized:

*I feel a bit unprepared* (…) (Interviewee 06)

(…)*. I feel a little weakened because I believe that there is still a lack of preparation, a lack of protocol, a lack of routine, a lack of much more knowledge* (…) (Interviewee 07)

*I have a huge difficulty in serving this type of patient due to unpreparedness, lack of training and lack of affinity with the mental health area* (…) (Interviewee 17)

The lack of knowledge in mental health and of skills in the approach and welcoming of patients and their families was also verbalized:

*I don’t find it easy because it requires a peculiar thing. I sometimes can’t understand the pathology very well. It’s difficult, for me it’s a little difficult.* (Interviewee 10)

*I have a lot of difficulty in this issue of dealing with psychiatric patients, because it is not an easy area, it is a very difficult area to deal with feelings, with emotions, with the psychological, with everything together and the family members as well* (…) (Interviewee 21)

From the TSs, it can be seen that the subjectivity and intersubjectivity of mental health care interfere substantially in the formation of SRs about this type of care, making it difficult to materialize this care into something practicable and applicable in the daily practice of an emergency service which, by its very nature, values objective care and quick results to the actions that are practiced.

### Category 3: Unveiling the Mental Health Care Provided by Nurses in the UPA

Taken from the lexicons derived from classes 1 and 5, it portrays how the reception and follow-up of cases requiring mental health care in the PCU occurs, as verbalized by one interviewee:

*We welcome this patient. If it is a simpler case, we refer this patient to a doctor’s office for a medical consultation* (…) *if it is an outbreak patient,* (…)*, the approach has to be different. We approach this patient, classify him as red or yellow, and send him to the emergency room. And then we have to have another approach that is a little more aggressive, because if this patient is having an outbreak, he puts our assistance at risk, our assistance, the team at risk, and even himself* (Interviewee 22)

It is noteworthy in this statement that the representational contents about a patient in crisis are linked to segregating, repressive practices and a threat to everyone’s safety. It can be seen that the professional is taken by the risk classification model and is not able to objectify a mental health care linked to the principles of BPR. The following excerpt, from another interviewee, exposes how the representations coexist with care models that reproduce the asylum practices:

(…) *we are not prepared to attend this patient, so he is treated with a lot of violence, with a lot of prejudice* (…).(Interviewee 12).

Physical, chemical and mechanical restraints are widely used as opposed to listening and welcoming, and often performed in an inadequate manner, without technical knowledge and with inadequate materials:

*The staff sometimes contains in an inappropriate way.* (…) *we don’t have the bands here suitable for containment* (…).(Interviewee 02)

*I believe that medication restraint and bed restraint should be more standardized, because we have several professionals* (…)*, the shift changes, the procedures change accordingly.* (Interviewee 04)

It can be noticed that one of the main concerns verbalized by the interviewees is focused on the performance of the technique itself, on how to make it more effective, and not on the patient as the object of care in the sense of creating strategies to alleviate his suffering. This situation is anchored in representations prior to the BPR in which the focus was on containing expressions of madness and silencing feelings and reactions. However, the existence of positive and successful initiatives by some nurses was also observed:

*I really enjoy talking, guiding the patient, trying to minimize the impact that the actions have on the patient and the family themselves. I feel good when I am successful* (Interviewee 09)

The following statements denote attempts to succeed in the care both to the patient with mental health needs and to his family, but permeated by incapacitating feelings:

(…) *sometimes we don’t seem to be able to provide all the care.* (…) *And not only the patient, there is the family part. The family member ends up feeling isolated because he perceives the looks from the other patients, even from the team* (…) (Interviewee 03)

(…) *in fact, we suffer together with the patient, patient and family.* (Interviewee 15)

The last statements denote the existence of a core where there is the ideal of a perspective of an individual and family care based on the principles of BPR that can originate and re-signify, at an individual and collective level, the SR of mental health care in this context.

## DISCUSSION

A social representation speaks as much as it shows, communicates as much as it expresses, produces and determines behaviors^(10)^. In light of this theory, it was possible to apprehend the perceptions of the participants about mental health care in the UPA through the identification of the existing languages and how this conjuncture marks the actions of the researched group.

The problem begins from the objectification of the important ignorance about the RAPs verbalized by the participants and, being it an element of articulation of the mental health care points and governed by guidelines that ensure a humanized, equitable and community-based care^(2)^, its non-recognition and the consequent disarticulation between services contributes significantly to a fragmented and unresolved assistance^(8)^.

It is noteworthy that the lack of knowledge about the current situation of the functioning of the RAPs in the country is, in fact, a reality and, with regard to the emergency network, there are even greater difficulties in the recognition of these services as its constituents^(9)^. In this context, it can be observed that such social reality is formed from the unfamiliar incorporated into the consensus universes of the research participants from the construction of the experienced reality^(15)^.

Another anchoring observed that supports the objectification of ignorance refers to the structural issues of the service to receive people with mental disorders, especially those in crisis, which does not provide a safe environment and the guarantee of secrecy, privacy and confidentiality of the patient^(6,16, 17, 18, 19, 20)^.

It became evident that between health professionals and the person with mental disorder there is a chasm whose depth is proportional to the non-identification with the mental health area, the negative feelings triggered and the lack of preparation, knowledge and skills reported by the interviewees.

The negative feelings are recurrent in the health professionals in these services, such as frustration and intimidation^(21)^, dissatisfaction^(22)^, discomfort^(7,21,22)^ and fear^(7,23)^. In some of the speeches we can observe that such feelings are anchored in previous negative experiences, which goes beyond the lack of training and suggests lack of personal preparation^(17)^. Considering that the SRs govern our relationship with the world and with others, guiding and organizing behaviors and social communications^(11)^, it is understood how such process is determinant in the practices performed, in this case in mental health care.

The non-appropriation of knowledge in mental health based on the principles of BPR and the remaining concepts of classical psychiatry make professionals anchor this care to outdated concepts and, added to the lack of established flows and protocols for this type of care, promote a gap between them and the users. The lack of specific knowledge and skills, experience and qualification in the area is recurrent among health professionals working in general urgencies and emergencies^(5–7,17–21,24–27)^, as well as the lack of specific protocols for the care of psychiatric urgencies and emergencies^(8,26)^.

As for the mental health care provided by nurses in the UPA, it is observed that the reception starts with risk classification based on the Manchester Protocol, in which the non-urgent or slightly urgent cases can be solved in the usual way as in any other non-psychiatric situation. Patients in outbreak are classified as urgent, very urgent or emergency, receive immediate care, but with a repressive character, which indicates that the workers of the UPA find in containment and medication ways to deal with such phenomena and appease the crisis^(28)^.

The interventions in psychic crisis guided by controlling and repressive actions reproduce the influence of the asylum model. The justification for the use of physical, mechanical and chemical restraint would be to preserve the safety of the staff, which is observed in other studies^(6,7,25,26)^. Since SRs, if viewed in a passive way, are apprehended as a reflection in individual or collective consciousness^(10)^, this allows explaining why obsolete concepts and practices based on the anchoring and objectification elements that form them are still reproduced.

Despite such reproductions, there are attempts by professionals to achieve the best possible care within the existing conditions, and to establish effective communication with both the user and his family. Theoretical and practical knowledge, communication skills, and a respectful attitude toward the patient are essential competence requirements for assessing people with mental disorders^(29)^. Satisfactory levels of knowledge promote increased confidence, positive attitudes, empathy and improvement in the quality of care provided^(5,30)^ seem to be the way to a care based on ethical and legal precepts and the realization of the rights of this public in health services, especially in emergency rooms.

Finally, the SR as elements that intervene in processes as varied as the dissemination and assimilation of knowledge, individual and collective development, definition of personal and social identities, expression of groups and social transformations^(11)^ are essential to elucidate the current situation of health practices and provide elements to remodel them in order to enable advances, which can be applicable to the care provided by nurses about the present object of study target of this research.

As limitations, we emphasize that these findings may not represent the object of study for other nurses in other PCU, given the dynamics of SR.

## CONCLUSIONS

This study allowed us to understand the SRs of nurses about mental health care developed in a PHU, as well as the conception of these professionals about the RAPs, showing many challenges and obstacles and some potentialities in the care model developed.

It was identified the scarcity of elements of anchoring and objectification about the RAPs, through the manifestation of little or no knowledge reported during the speeches, which favors a fragmented assistance between the points of care and harm in the resoluteness of cases and continuity of care. Regarding the professional-patient relationship, anchoring elements associated with previous negative experiences in mental health care were observed, which seems to cause a kind of permanent trauma in some professionals. Most of the time, nursing care is permeated by cognitive barriers that hinder adequate mental health care.

Such findings are unprecedented in the locality studied and capable of promoting a (re)signification of the work process of nurses in this scenario, qualifying their work, which consequently promotes the improvement of the work process in the UPA as a RAPs care point.

## References

[1] Brasil. Ministério da Saúde. Secretaria de Atenção à Saúde (2013). Departamento de Atenção Básica. Saúde mental [Internet].

[2] Brasil. Ministério da Saúde. Portaria nº 3088, de 23 de dezembro de 2011. (2011). Institui a Rede de Atenção Psicossocial para pessoas com sofrimento ou transtorno mental e com necessidades decorrentes do uso de crack, álcool e outras drogas, no âmbito do Sistema Único de Saúde (SUS). Diário Oficial da União [Internet].

[3] Cruz KDF, Guerrero AVP, Scafuto J, Vieira N (2019). Atenção à crise em saúde mental: um desafio para a reforma psiquiátrica brasileira. Rev NUFEN..

[4] Souza BS, Pio DAM, Oliveira GTR (2021). Perspectivas de usuários em sofrimento psíquico sobre um Serviço de Pronto Atendimento. Psicologia (Cons Fed Psicol).

[5] Koning KL, Mcnaught A, Tuffin K (2018). Emergency Department staff beliefs about self-harm: a thematic framework analysis. Community Ment Health J..

[6] Macedo MM, Souza J, Almeida LY, Vedana KGG, Santos MA, Miasso AI (2018). Visits to an Emergency Department by children and adolescents with substance-related disorders and the perceptions of nursing professionals. Child Youth Serv Rev..

[7] Daggenvoorde TH, van Klaren JM, Gijsman HJ, Vermeulen H, Goossens PJJ (2021). Experiences of Dutch ambulance nurses in emergency care for patients with acute manic and/or psychotic symptoms: a qualitative study. Perspect Psychiatr Care..

[8] Oliveira LC, Silva RAR (2017). Knowledge and practices in urgent and emergency psychiatric care.. Rev Enferm UERJ..

[9] Sampaio ML, Bispo JP (2021). Rede de Atenção Psicossocial: avaliação da estrutura e do processo de articulação do cuidado em saúde mental. Cad Saude Publica..

[10] Moscovici S (1978). A representação social da psicanálise.

[11] Jodelet D (2001). As representações sociais.

[12] Souza VRS, Marziale MHP, Silva GTR, Nascimento PL (2021). Tradução e validação para a língua portuguesa e avaliação do guia COREQ. Acta Paul Enferm..

[13] Fontanella BJB, Luchesi BM, Saidel MGB, Ricas J, Turato ER, Melo DG (2011). Amostragem em pesquisas qualitativas: proposta de procedimentos para constatar saturação teórica. Cad Saude Publica..

[14] Camargo BV, Justo AM (2013). IRAMUTEQ: um software gratuito para análise de dados textuais. Temas Psicol..

[15] Sá CP, Spink MJ (2004). O conhecimento no cotidiano: as representações sociais na perspectiva da psicologia social.

[16] Chou HJ, Tseng KY (2020). The experience of emergency nurses caring for patients with mental illness: a qualitative study. Int J Environ Res Public Health..

[17] Pereira LP, Duarte MLC, Eslabão AD (2019). Care for people with psychiatric comorbidity in a general emergency unit: vision of the nurses. Rev Gaúcha Enferm..

[18] Broadbent M, Moxham L, Dwyer T (2020). Understanding nurses perspectives of acuity in the process of emergency mental health triage: a qualitative study. Contemp Nurse..

[19] Dombagolla MHK, Kant JÁ, Lai FWY, Hendarto A, Taylor DMD (2019). Barriers to providing optimal management of psychiatric patients in the emergency department (psychiatric patient management). Australas Emerg Care..

[20] Fontão MC, Rodrigues J, Lino MM, Lino MM, Kempfer SS (2018). Nursing care to people admitted in emergency for attempted suicide. Rev Bras Enferm.

[21] Beks H, Healey C, Schlicht K (2018). ‘When you’re it’: a qualitative study exploring the rural nurse experience of managing acute mental health presentations. Rural Remote Health..

[22] Molina-Mula J, González-Trujillo A, Simonet-Bennassar M (2018). Emergency and mental health nurses’ perceptions and attitudes towards alcoholics. Int J Environ Res Public Health..

[23] Usher K, Jackson D, Woods C, Sayers J, Kornhaber R, Cleary M (2017). Safety, risk, and aggression: health professionals: experiences of caring for people affected by methamphetamine when presenting for emergency care. Int J Ment Health Nurs..

[24] Ács A, Mészáros J, Balogh Z (2020). Egészségügyi szakdolgozók mentális zavarokkal kapcsolatos ismereteinek és a betegekkel szembeni attitűdjének vizsgálata. Orv Hetil..

[25] Fry M, Abrahamse K, Kay S, Elliott RM (2019). Suicide in older people, attitudes and knowledge of emergency nurses: a multi-centre study. Int Emerg Nurs..

[26] Maina R, Bukusi D, Njuguna SK, Kumar M (2018). Gaps in suicide assessment and management among accident and emergency nurses in Kenyatta National Hospital: a qualitative study. Glob Soc Welf..

[27] Holmberg M, Hammarbäck S, Andersson H (2020). Registered nurses’ experiences of assessing patients with mental illness in emergency care: a qualitative descriptive study. Nord J Nurs Res..

[28] Homercher BM, Volmer A (2021). Interlocuções entre acolhimento e crise psíquica: percepção dos trabalhadores de uma unidade de pronto-atendimento. Physis..

[29] Andersson H, Carlsson J, Karlsson L, Holmberg M (2020). Competency requirements for the assessment of patients with mental illness in somatic emergency care: a modified Delphi study from the nurse’s perspective. Nord J Nurs Res..

[30] Ngune I, Hasking P, McGough S, Wynaden D, Janerka C, Rees C (2021). Perceptions of knowledge, attitude and skills about non-suicidal self-injury: a survey of emergency and mental health nurses. Int J Ment Health Nurs..

